# Nursing consultation based on self-care supported by people with Diabetes *Mellitus:* an experience report

**DOI:** 10.1590/0034-7167-2024-0277

**Published:** 2025-12-08

**Authors:** Gabrielly Segatto Brito, Rosilene Rocha Palasson, Sonia Silva Marcon, Elen Ferraz Teston

**Affiliations:** I Universidade Federal de Mato Grosso do Sul. Campo Grande, Mato Grosso do Sul, Brazil; II Universidade Estadual de Maringá. Maringá, Paraná, Brazil

**Keywords:** Self Care, Primary Health Care, Diabetes *Mellitus*, Nursing, Self-Management., Autocuidado, Atención Primaria de Salud, Enfermería de Consulta, Diabetes Mellitus, Enfermería, Automanejo.

## Abstract

**Objectives:**

to report the experience of implementing nursing consultation based on supported self-care for people with type 2 diabetes *mellitus*.

**Methods:**

an experience report regarding the implementation of nursing consultation based on supported self-care for people with diabetes. The intervention was preceded by the organization of instruments based on the adopted theoretical framework and by defining the objective of the in-person consultations and telephone monitoring.

**Results:**

the instrument used to conduct the consultations consisted of four printed forms, the first intended for initial consultation, the second for follow-up consultation, the third for telephone monitoring, and the fourth for the final research consultation.

**Final Considerations:**

the implementation of nursing consultation based on supported self-care enables a structured and continuous approach that favors people’s engagement in self-care, strengthening nurses’ role in educational and motivational support for people with chronic conditions.

## RESUMEN

**Objetivos:**

reportar la experiencia de implementación de una consulta de enfermería basada en el autocuidado con apoyo para personas con diabetes *mellitus* tipo 2.

**Métodos:**

reporte de experiencia sobre la implementación de una consulta de enfermería basada en el autocuidado con apoyo para personas con diabetes. La intervención fue precedida por la organización de instrumentos basados en el marco teórico adoptado y la definición del objetivo de las consultas presenciales y el seguimiento telefónico.

**Resultados:**

el instrumento utilizado para realizar las consultas consistió en cuatro formularios impresos: el primero destinado a la consulta inicial, el segundo a la consulta de seguimiento, el tercero al seguimiento telefónico y el cuarto a la consulta final de investigación.

**Consideraciones Finales:**

la implementación de una consulta de enfermería basada en el autocuidado con apoyo permite un enfoque estructurado y continuo que favorece la participación de las personas en el autocuidado, fortaleciendo el rol de las enfermeras en el apoyo educativo y motivacional a las personas con enfermedades crónicas.

## INTRODUCTION

Nursing consultation (NC) is a care organization activity in which nursing professionals have the autonomy to develop comprehensive care strategies aimed at promoting health, early diagnosis and treatment, in addition to preventing avoidable situations involving the person, family or community. Its performance is legally supported by the Professional Practice Law, which legitimizes it as an activity exclusive to nurses^(1,2)^.

Consultation should include the nursing process stages, based on theoretical models that encourage individuals’ active participation in self-care^(2)^.

The Brazilian National Primary Care Policy (In Portuguese, *Política Nacional da Atenção Básica* - PNAB) assigns several responsibilities to nurses in the context of Primary Health Care (PHC), including conducting NC, requesting additional tests and prescribing medications. It is essential that these actions are carried out in strict accordance with nursing legal guidelines and in line with protocols or technical regulations in force at the national, state and municipal levels^(3)^.

However, although NC is an essential activity in PHC, it is not always carried out in a systematic manner and is often limited to specific and specific care. This fragmentation compromises the continuous monitoring of patients, making it difficult to identify complications early, ensure adherence to treatment and implement personalized interventions, which is at odds with the Chronic Care Model (CCM) assumptions^(4)^.

For individuals with type 2 diabetes mellitus (T2DM), the lack of structured monitoring can lead to difficulties in managing the health condition, increased risk of chronic complications, and reduced adherence to lifestyle changes. The lack of systematization can also create gaps in communication between professionals and patients, reducing the impact of self-care guidelines and impairing individuals’ autonomy in managing the disease. A study carried out in southern Brazil identified that the main challenges for implementing NC are related to nurses’ work process, marked by overload, accumulation of administrative and assistance functions, lack of time, deficit of human and material resources, and high demand from users in health services^(2)^.

In addition to these obstacles, care is still centered on the biomedical model, with little or no encouragement for self-care actions. Thus, to live up to PHC professionals’ commitment to promoting patient-centered health practices geared toward positive health outcomes as proposed in the PNAB, it is essential to adopt approaches that consider a person in their individuality, complexity, and comprehensiveness^(3)^.

Regarding self-care practices for coping with T2DM, gaps were identified in studies whose results provide sufficient support to health professionals so that they can improve educational actions and interventions that are carried out in the routine of services, and in the case of nursing professionals, that the results can, for instance, favor the implementation of a NC that is truly effective for people with chronic conditions^(4,5)^.

Care for these individuals should be guided by actions that encourage, for instance, awareness of the importance of their individual behaviors and their role in the proper management of the chronic condition and in changing habits. In this context, CCM proposes strategies aimed at self-management of health, emphasizing the active role of individuals in controlling the disease. A such strategy is supported self-care, which expands this approach by providing ongoing support for individuals to develop autonomy in managing diabetes. In addition to strengthening the bond between professional and patient, this model allows individuals to acquire knowledge and skills to make informed decisions about their health, promoting more effective and sustainable care^(5)^.

This, in turn, aims to value the central role of individuals with chronic conditions, preparing and empowering them to take care of themselves and manage their health, with professional support^(5)^. In this context, as recommended by CCM, welcoming and strengthening the bond favors the identification of problems, the joint establishment between health professionals and individuals, and goals to be achieved for adequate control of the health condition^(5)^.

Known as the 5 “A’s” methodology, supported self-care consists of five interrelated pillars that are used to support a person in the process of managing their own health and structuring the care plan. These pillars are part of the “5 A’s” methodology, which are ask, advise, assess, assist, and arrange^(5)^.

In this regard, systematic care actions, focusing on promoting self-care for individuals with T2DM, are considered fundamental to achieving positive results in the chronic condition management. Given this initial contextualization, the question is: how can the assumptions of supported self-care support the NC systematization for people with T2DM?

## OBJECTIVES

To report the experience of implementing NC based on supported self-care for people with T2DM.

## METHODS

This is a descriptive study, in the form of an experience report, prepared based on the implementation of a nursing intervention proposed by a funded matrix research project, approved by the Research Ethics Committee of a federal university in the Brazilian Central-West region.

**Figure 1:**
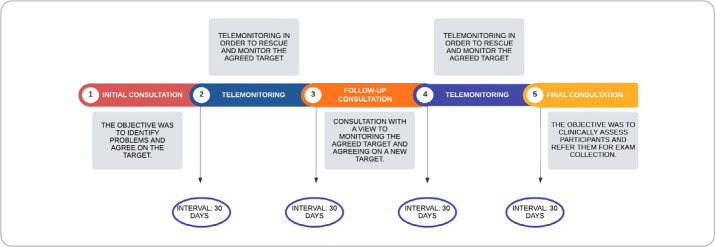
Illustration of the schedule of consultations carried out

The proposed intervention was structured around three monthly in-person NCs, interspersed with two monthly telephone calls, the objective of which was to monitor the goals agreed upon during NCs (Figure 1).

In order to organize and systematize the intervention, the main researcher constructed an instrument (divided into four sections) to conduct the intervention and collect research data, based on the theoretical framework adopted and pre-existing models in the supported self-care exercise booklet^(5)^.

Section 1 of the instrument (used in the first NC) was composed of user characterization variables, clinical data, health conditions, health behaviors, general physical and foot assessment, care plan and, finally, goal agreement contract.

User characterization was supported by basic information, such as age, marital status, education, current occupation, who they live with, and family history of diabetes. Clinical data and health conditions are composed of information regarding a person’s knowledge regarding diabetes and its management, such as recognition of hypoglycemia and hyperglycemia, recognition of complications caused by diabetes, whether there have been hospitalizations in the last year, whether they demonstrate knowledge of medications and their regimen, whether they use insulin, in addition to a field to indicate whether there are comorbidities.

As for health behaviors, the instrument addresses questions about smoking, alcohol consumption, physical activities, leisure activities and stress management. If so, it is necessary to inform the frequency of each of them. Moreover, the questionnaire included an open-ended question titled “Tell me about your diet”, such as “How do you consider your diet? Is there anything that needs to be changed?”, which aims to encourage self-reflection on the habit. The next item in the instrument is intended to provide information on whether or not a user receives help to prepare meals.

The final part of the “health behaviors” section addresses questions about the ability and frequency of checking blood glucose, participation in the Hiperdia group, a person’s perception of their health status, their weight and an open-ended question: “Please assess the behaviors you have that you believe influence your health care in relation to diabetes”.

This question aims to assess a person’s knowledge about their lifestyle and health condition, as well as the degree of motivation and confidence to adopt healthier behaviors. Furthermore, there is a field for professionals to identify whether or not a person has family support as well as the stage of motivation for change at that time.

The physical examination section consisted of a cardiorespiratory examination, height and weight measurement for Body Mass Index classification, abdominal circumference, blood pressure and capillary blood glucose measurement, in addition to foot assessment.

The assumptions of supported self-care, one of the pillars of CCM, are based on the 5A’s methodology (ask, advise, assess, assist, and arrange)^(5)^. Thus, section 1 of the instrument provides support for nurses to assess individuals’ beliefs, values, knowledge, behaviors and identify objective and subjective health needs from professionals’ and peoples’ perspective.

The survey of problems within NC was carried out seeking to identify the main difficulties reported by participants in relation to self-care and habits adopted through a welcoming approach, allowing patients to feel comfortable expressing their difficulties.

Also, in section 1 of the instrument, there is space for recording the agreement made between the professional and the user through the establishment of goals, based on the “problem” selected by users. This agreement involves the behaviors and activities that will be undertaken in the coming days and contributes to a person’s accountability for the process and its results. To do this, a person must be able to describe what they will do, for how long, how many times a day or week, where/when and how in a specific and realistic manner (Figure 1).

**Figure 2:**
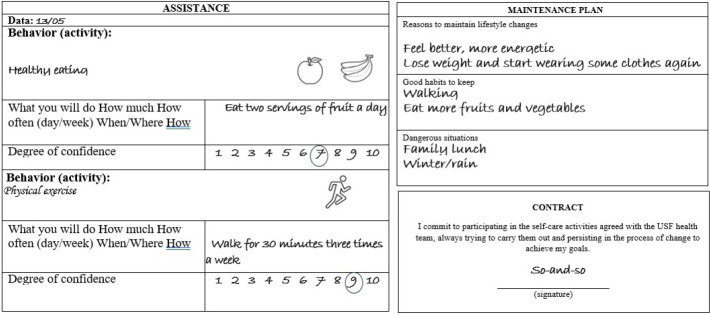
Illustration of care plan, maintenance plan and contract

Furthermore, there is the representation of a contract, which the patient must sign, with the aim of encouraging the person’s accountability for the process and its results (Figure 2).

Section 2 of the instrument consisted of questions about monitoring the agreed goals that were used in the telephone calls between in-person consultations. This was based on “assist” and “arrange”, which aims to monitor solutions implemented through the maintenance plan. The telephone monitoring began by recalling the goal that had been agreed upon in the in-person consultation. The user described the difficulties and facilities that were faced during the period, and the identification of barriers was carried out, and the possibilities for overcoming them were discussed.

Section 3 was composed of the recording structure of consultation 1 to be used for new agreements, with the objective of “arranging” and “assisting”. When previously established goals were achieved, individuals were asked to indicate another problem or difficulty to be overcome and the possible actions to overcome it.

Section 4 aimed to perform the final clinical assessment of the intervention as well as to encourage lasting behavioral change in participants.

The instrument was organized into four sections with the aim of systematizing the care process to ensure continuous and structured monitoring of participants’ progress throughout the intervention.

## RESULTS

### Instrument organization

The instruments used were constructed by research nurses, based on guidelines contained in the “Supported Self-Care: a health professional’s manual” booklet, consisting of four forms for conducting and recording NCs (Chart 1).

**Chart 1:** Instrument structuring

Instrument	Objective
**Initial nursing consultation**	Collect identification, clinical, behavioral and social data for situational diagnosis and care planning.
**Follow-up consultation**	Monitor adherence to agreed goals, review the self-care plan and renegotiate strategies.
**Telephone monitoring**	Assess the progress of behavioral changes and offer support for difficulties encountered.
**Final consultation**	Compare initial and final data, assessing the impacts of the intervention on participants’ health.

Based on the assumptions of supported self-care, it aims to reorganize care practices in contrast to the predominant biomedical care that has not shown effective impacts in the field of chronic conditions. It is based on the premise of empowering and preparing individuals for self-management of their health condition^(5)^.

At the end of the instrument development, a discussion was held between members of the research group and professionals who, during the investigation period, worked in the health service, with the aim of verifying the possibility of its application. After discussion, adjustments were necessary to the instrument due to its length.

### Use of the instrument to systematize nursing consultation

Consultations, which lasted an average of fifty minutes, involved the application of auxiliary research questionnaires and anthropometric data collection. During this process, the main researcher noticed that participants felt strange when asked in detail about their lifestyle habits, when they were listened to and encouraged to reflect on their own habits.

The NC systematization, using the premises of supported self-care, allowed the recording and counseling according to each participant’s individuality and motivation, which favored the acceptance and recognition of the need for behavior change, which could be verified in participants’ statements during telemonitoring and in in-person meetings at the unit.

**Figure 3:**
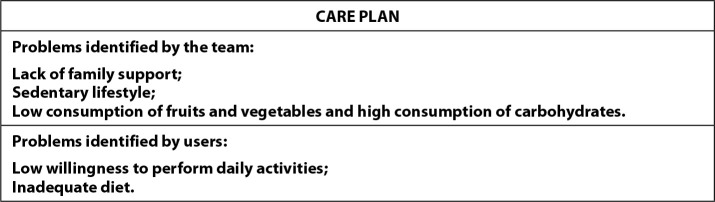
Illustration of the care plan according to the problems identified by users and professionals

The structured instrument application facilitated the assessment of current behaviors, favoring the identification by users of habits that needed to be changed. Based on self-assessment of current behavior, it was possible for the researcher to recognize details about participants’ routine and clarify possible doubts, which enhances engagement in the care process. Figure 3 illustrates the care plan with problems identified by the user and professional partnership.

It is important to emphasize that, based on these recognitions, nurses organize “advise” using the most effective strategies, supporting self-care and the process of behavior change centered on the person, their availability and their need.

Initially, participants demonstrated difficulty in reflecting on the need for behavioral change. Furthermore, there was a belief that medication treatment would be sufficient for managing the chronic condition, due to their familiarity with the biomedical model. This resulted in an initial resistance to non-medication treatment. However, after observing clinical results resulting from behavioral changes, participants began to recognize the positive impact of these changes.

**Figure 4:**
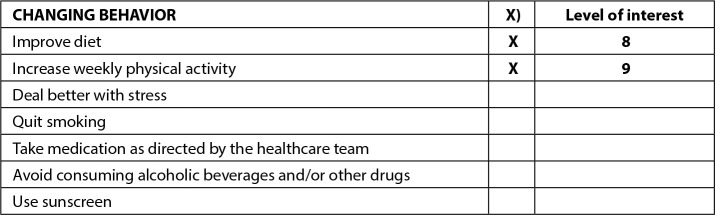
Illustration of the degree of interest in changing

It is important to highlight the need to identify the level of confidence that a person describes when proposing a certain goal, as shown in Figure 4, since the level of confidence shows how much people believe they are capable of achieving their goals and sustaining them. To do this, they need to assess the effort required to act differently. Therefore, immediately after agreement was reached, it was necessary to give a score from zero to ten for confidence in being able to execute the plan. The last item present in the first printed version of the instrument is the contract (Figure 1).

When making agreements, professionals explain the purpose of the contract and the importance of signing it at this time. Its character is moral, reinforcing the commitment made to oneself for change^(6)^. To this end, there was a question regarding the positive and negative aspects of the change process. Solutions that are appropriate for the purpose must be reinforced, and those that are inadequate or more difficult must be reviewed and reorganized.

Furthermore, as a task is completed, others are chosen so that problem-solving skills progressively develop with greater spontaneity and autonomy. In all consultation forms, there are fields for performing a physical examination so that parameters can be compared.

**Figure 5:**
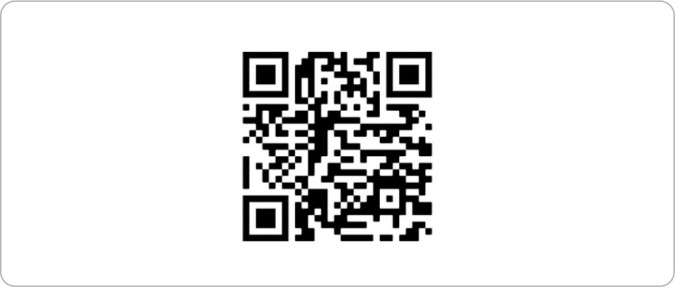
QR Code to view the developed instrument

Systematization, through a specific instrument (Figure 5), facilitated the conduct of NC and favored the recording of information, thus ensuring accurate documentation, essential for interprofessional communication and continuity of care. Through consultation systematization, it was possible to continuously monitor participants’ evolution over time, allowing adjustments to care plan as necessary.

At the end of the intervention, differences were observed in self-perception regarding the need for behavioral change, in addition to improvements in clinical parameters. These changes were verified in participants’ speech, in laboratory data and in anthropometric data collected throughout the research period.

In this scenario, nurses must use strategies to achieve this. Initial goals need to be tangible, otherwise they may feel unmotivated and give up, which makes this task a challenge.

Thus, active listening is essential to capture explicit complaints and identify problems that may not be mentioned directly, and identifying patients’ difficulties is essential to offer effective and personalized guidance. Furthermore, during physical examination and interaction with patients, nurses can identify signs that indicate difficulties in self-care, such as inadequate hygiene, skin changes and facial expressions that demonstrate discomfort or insecurity.

## DISCUSSION

To ensure the quality of nursing care for people with diabetes, nurses must monitor them by carrying out NC, based on the nursing process, which aims to understand a person’s previous history during subjective data collection, the identification of existing problems, the development of a targeted care plan, the prioritization of nursing diagnoses, the expected results and their respective evolution^(6)^.

Health and nursing practices still persist with characteristics of the biomedical, mechanistic model, focused on professionals, rather than health action that is capable of understanding the most comprehensive needs of users and families, in a context that aims at individualized care^(2,5)^.

Participant characterization aims to identify the a person’s specificities and individual needs so that actions are in accordance with social needs, in addition to intervening in reality in an articulated and co-responsible manner^(7)^. Studies show that NC and educational actions based on supported self-care impact health self-management, increase the stimulus for self-care and contribute to behavior change^(2,4,5)^.

Therefore, it is necessary to implement individual and collective actions that can stimulate awareness among individuals in recognizing their role in the adequate management of the disease in the context of health services that make up PHC and maintaining quality of life^(2,5)^. It is worth noting that these actions take time, mainly because professionals need to understand and learn to work with users’ subjectivities, which is based on a relationship of trust, and because patients need to understand that professionals provide support in the process of changing behavior and engaging with their health condition. To this end, nurses can use techniques such as motivational interviews, setting realistic goals, positive feedback and ongoing support for patients. The use of self-care diaries and periodic monitoring are also effective strategies for maintaining motivation in the long term.

In 1982, researchers described five different motivational stages for behavior change: pre-contemplation; contemplation; preparation; action; and maintenance. As a process, it is dynamic, changing over time and in different circumstances. Motivation, therefore, is an internal stage of disposition for change and is influenced by external factors. Identifying the stage of change is important because it allows us to know how motivated a person is to effectively change^(8)^. Motivation for change encompasses the stages that need to be identified so that the most effective interventions can be implemented. The assessment of these stages is crucial for choosing the most appropriate and effective therapeutic strategies for increasing and maintaining motivation, favoring effective behavior change.

Thus, NC, when carried out systematically, constitutes the ideal scenario for encouraging self-care, contributing to behavior change. Although it is recommended as a comprehensive activity of PHC, NC for people with chronic conditions is still not systematized in most health services in Brazil and, even when carried out, presents gaps in its implementation and execution^(9)^.

Nursing care for people with chronic conditions needs to be focused on a health education process that helps individuals to live with and recognize their role in managing the chronic condition, making them co-responsible for their care^(2,4)^. A meta-analysis study, carried out from six randomized clinical trials, which aimed to determine the effectiveness of self-management strategies for individuals with T2DM, showed a reduction in HbA1C in the intervention groups, indicating an improvement in treatment by contributing to quality of life and self-care behavior^(10)^.

In the Brazilian context, the use of supported self-care during NC was tested by a study carried out in southern Brazil, which demonstrated that, after assessing the individual intervention based on supported self-care (composed of NC and telephone monitoring), there was a significant increase in knowledge and positive attitude towards the disease, the impact on quality of life and adherence to self-care activities^(2)^.

However, some challenges permeate the implementation of NC with this approach, such as the difficulties patients have in reflecting on their lifestyle, because this process takes time and requires a certain degree of self-knowledge and understanding of their own daily behavior. Individuals need to realize that medication alone will not solve the problem, and this resistance is slow, in addition to being influenced by social determinants, socioeconomic, educational and cultural factors that influence the perception and adherence to behavioral changes. For instance, individuals with a low level of education may have difficulty understanding self-care guidelines, while economic factors may limit access to adequate nutrition and resources for glycemic monitoring.

To overcome these challenges, it would be essential to implement standardized guidelines for NC, continuous training of nursing professionals and strengthening of public policies that encourage systematic and accessible consultation for the entire population.

### Study limitations

As a limitation, the non-use of a standardized language system during consultation stands out. It is recognized that the use of this is fundamental for the nursing process and consultation systematization, highlighting the need for future research that can address it in a more comprehensive way. However, the results generated from this study can contribute to improving the quality of care provided by nurses, in addition to identifying various aspects of people’s health, including not only physical aspects, but also emotional, social, family and psychological aspects, promoting a comprehensive approach to care.

### Impacts on nursing care practice

The importance of using an auxiliary instrument for planning, systematizing and recording care actions during NC for people with diabetes is highlighted. This practice not only facilitates the organization and effectiveness of interventions, but also promotes safe and higher quality care, contributing to health promotion and chronic condition management. Thus, the implementation of systematization strategies, such as the one carried out in the present study, can contribute to optimizing the care process and achieving more positive clinical and well-being results for this specific population.

## FINAL CONSIDERATIONS

The experience of NC based on supported self-care emerges as a tool capable of promoting a user-centered intervention, enabling individuals’ autonomy while maintaining the caring essence inherent to nursing practice. Systematizing and recording this process not only facilitates adherence to necessary changes in lifestyle, but also promotes learning and increases self-efficacy to deal with everyday challenges. Thus, the relevance of this approach in the provision of nursing care is evident, as it not only strengthens the therapeutic relationship, but also enhances positive results in promoting individuals’ health and well-being.

## Data Availability

The research data are available within the article.
