# Supplementary material for: Nursing consultation based on self-care supported by people with Diabetes Mellitus: an experience report
Source: Rev Bras Enferm. 2025 Dec 8;78(6):e20240277. doi: 10.1590/0034-7167-2024-0277 (PMC12707612; doi:10.1590/0034-7167-2024-0277)
Supplement: Translation

# Translation: Consulta de enfermagem baseada no autocuidado apoiado às pessoas com Diabetes *Mellitus:* relato de experiência

**DOI:** 10.1590/0034-7167-2024-0277pt

**Authors:** Gabrielly Segatto Brito, Rosilene Rocha Palasson, Sonia Silva Marcon, Elen Ferraz Teston

**Affiliations:** I Universidade Federal de Mato Grosso do Sul. Campo Grande, Mato Grosso do Sul, Brasil; II Universidade Estadual de Maringá. Maringá, Paraná, Brasil

**Keywords:** Autocuidado, Atenção Primária à, Saúde, Diabetes *Mellitus*, Enfermagem, Autogestão.

## Abstract

**Objetivos:**

relatar a experiência de implementar a consulta de enfermagem baseada no autocuidado apoiado às pessoas com Diabetes *Mellitus* do tipo 2.

**Métodos:**

relato de experiência referente à implementação da consulta de enfermagem baseada no autocuidado apoiado às pessoas com diabetes. A intervenção foi precedida da organização dos instrumentos, a partir do referencial teórico adotado, e da definição do objetivo das consultas presenciais e do monitoramento telefônico.

**Resultados:**

o instrumento utilizado na condução das consultas foi composto de quatro impressos, sendo o primeiro destinado à consulta inicial, o segundo, à consulta de acompanhamento, o terceiro, ao monitoramento telefônico, e o quarto, à consulta final da pesquisa.

**Considerações Finais:**

a implementação da consulta de enfermagem baseada no autocuidado apoiado possibilita uma abordagem estruturada e contínua que favorece o engajamento das pessoas no autocuidado, fortalecendo a atuação do enfermeiro no suporte educacional e motivacional às pessoas com condições crônicas.

## INTRODUÇÃO

A consulta de enfermagem (CE) é uma atividade de organização do cuidado em que o profissional enfermeiro dispõe de autonomia para elaborar estratégias de cuidado integral com vistas à promoção da saúde, diagnóstico e tratamento precoce, além da prevenção de situações evitáveis que envolvem a pessoa, família ou comunidade. Sua realização é sustentada legalmente pela Lei do Exercício Profissional, que a legitima como uma atividade privativa do enfermeiro^(1,2)^.

A consulta deve incluir as etapas do processo de enfermagem, fundamentadas em modelos teóricos que incentivem a participação ativa do indivíduo no autocuidado^(2)^.

A Política Nacional da Atenção Básica (PNAB) atribui diversas responsabilidades ao enfermeiro no contexto da Atenção Primária à Saúde (APS), entre as quais estão a condução da CE, a solicitação de exames complementares e a prescrição de medicamentos. É fundamental que essas ações sejam realizadas em estrita conformidade com as diretrizes legais da profissão e em consonância com os protocolos ou normativas técnicas vigentes no âmbito nacional, estadual e municipal^(3)^.

Contudo, embora a CE seja uma atividade essencial na APS, sua realização nem sempre ocorre de forma sistematizada e, muitas vezes, restringe-se a atendimentos pontuais e específicos. Essa fragmentação compromete o acompanhamento contínuo do paciente, dificultando a identificação precoce de complicações, a adesão ao tratamento e a implementação de intervenções personalizadas, o que destoa dos pressupostos do Modelo de Atenção às Condições Crônicas (MACC)^(4)^.

Para indivíduos com Diabetes *Mellitus* do tipo 2 (DM2), a falta de um acompanhamento estruturado pode levar a dificuldades no manejo da condição de saúde, aumento do risco de complicações crônicas, além de menor adesão às mudanças no estilo de vida. A ausência de sistematização também pode gerar lacunas na comunicação entre profissionais e pacientes, reduzindo o impacto das orientações sobre autocuidado e prejudicando a autonomia do indivíduo no manejo da doença. Estudo realizado no sul do Brasil identificou que os principais desafios para efetivação da CE estão relacionados ao processo de trabalho do enfermeiro, marcado por sobrecarga, acúmulo de funções administrativas e assistenciais, falta de tempo, déficit de recursos humanos e materiais, e grande demanda de usuários nos serviços de saúde^(2)^.

Além desses entraves, a assistência ainda é centrada no modelo biomédico, com pouco ou nenhum estimulo às ações de autocuidado. Desse modo, para fazer jus ao compromisso dos profissionais da APS com a promoção de uma prática de saúde centrada no paciente e orientada para resultados positivos de saúde como proposto na PNAB, é imprescindível a adoção de abordagens que considerem a pessoa em sua individualidade, complexidade e integralidade^(3)^.

No que se refere às práticas de autocuidado para o enfretamento do DM2, foram identificadas lacunas de estudos cujos resultados deem subsídios suficientes aos profissionais de saúde de forma que eles possam aprimorar as ações educacionais e as intervenções que são realizadas na rotina dos serviços, e no caso dos profissionais enfermeiros, que os resultados possam, por exemplo, favorecer a realização de uma CE que seja deveras efetiva às pessoas com condições crônicas^(4,5)^.

O cuidado a essas pessoas deve ser direcionado por ações que estimulem, por exemplo, a conscientização sobre a importância de seus comportamentos individuais e de seu protagonismo no manejo adequado da condição crônica e na mudança de hábitos. Nesse contexto, o MACC propõe estratégias voltadas para o autogerenciamento da saúde, enfatizando o papel ativo da pessoa no controle da doença. Uma dessas estratégias é o autocuidado apoiado, que amplia essa abordagem ao fornecer suporte contínuo para que as pessoas desenvolvam autonomia no gerenciamento do diabetes. Além de fortalecer o vínculo entre profissional e paciente, esse modelo permite que os indivíduos adquiram conhecimentos e habilidades para tomar decisões informadas sobre sua saúde, promovendo um cuidado mais efetivo e sustentável^(5)^.

Esse, por sua vez, tem como propósito valorizar o papel central dos indivíduos com condições crônicas, preparando-os e emponderando-os para cuidar de si e gerenciar sua saúde, com apoio profissional^(5)^. Nesse contexto, tal como preconizado pelo MACC, o acolhimento e o fortalecimento do vínculo favorecem a identificação de problemas, o estabelecimento conjunto entre os profissionais de saúde e os indivíduos, e metas a serem alcançadas para um controle adequado da condição de saúde^(5)^.

Conhecido como a metodologia dos 5 “As”, o autocuidado apoiado é constituído por cinco pilares inter-relacionados que são utilizados para apoiar a pessoa no processo de gerenciamento de sua própria saúde e na estruturação do plano de cuidados. Esses pilares integram a metodologia dos “5 As”, que são avaliação, aconselhamento, acordo assistência e acompanhamento^(5)^.

Nesse sentido, ações de assistência de forma sistematizada, com foco na promoção do autocuidado pelo indivíduo com DM2, são consideradas fundamentais para alcançar resultados positivos no manejo e gestão da condição crônica. Diante dessa contextualização inicial, questiona-se: como os pressupostos do autocuidado apoiado podem embasar a sistematização da CE às pessoas com DM2?

## OBJETIVOS

Relatar a experiência de implementar a CE baseada no autocuidado apoiado às pessoas com DM2.

## MÉTODOS

Estudo descritivo, na modalidade de relato de experiência, elaborado a partir da implementação de uma intervenção de enfermagem proposta por uma pesquisa matricial financiada, aprovada pelo Comitê de Ética em Pesquisa de uma universidade federal da região Centro-Oeste brasileira.

A intervenção proposta foi estruturada a partir de três consultas de enfermagem presenciais, mensais, intercaladas com duas ligações telefônicas, também mensais, cujo objetivo era o monitoramento das metas pactuadas durante as CEs (Figura 1).

**Figura 1:** Ilustração do cronograma das consultas realizadas

Com o intuito de organizar e sistematizar a intervenção, a pesquisadora principal construiu um instrumento (dividido em quatro seções) para condução da intervenção e coleta de dados da pesquisa, a partir do referencial teórico adotado e dos modelos pré-existentes no caderno de exercício do autocuidado apoiado^(5)^.

A seção 1 do instrumento (utilizada na primeira CE) foi composta por variáveis de caracterização do usuário, dados clínicos, condições de saúde, comportamentos de saúde, exame físico geral e dos pés, plano de cuidados e, por fim, o contrato de pactuação de meta.

A caracterização do usuário foi subsidiada por informações básicas, como idade, estado civil, escolaridade, ocupação atual, com quem vive e histórico de diabetes na família. Os dados clínicos e condições de saúde são compostos por informações referentes aos conhecimentos da pessoa em relação ao diabetes e seu manejo, como reconhecimento de hipoglicemia e hiperglicemia, reconhecimento de complicações causadas pelo diabetes, se houve internações no último ano, se demonstra saber quais medicações e esquema de uso delas, se faz uso de insulina, além de um campo para marcar se há a presença de comorbidades.

Em relação aos comportamentos de saúde, o instrumento aborda questões sobre tabagismo, etilismo, atividades físicas, de lazer e de manejo do estresse, em caso positivo, necessário informar a frequência destinada a cada um deles. Além disso, fez parte do questionário uma questão aberta com o título “Fale-me sobre sua alimentação”, como “Como você considera sua alimentação? Tem algo que precisa ser mudado?”, que tem como objetivo instigar a autorreflexão sobre o hábito. O próximo item do instrumento se destina à informação sobre o usuário receber ou não auxílio para preparar as refeições.

A parte final da seção “comportamentos de saúde” aborda questões sobre a capacidade e frequência de verificação de glicemia, participação no grupo Hiperdia, a percepção da pessoa sobre seu estado de saúde, seu peso e uma questão aberta: “Faça uma avaliação sobre os comportamentos que você tem e que você acredita que influenciam o seu cuidado à saúde em relação ao diabetes”.

Essa questão tem como objetivo avaliar o conhecimento da pessoa sobre seu estilo de vida e sua condição de saúde, bem como o grau de motivação e confiança para assumir comportamentos mais saudáveis. Além disso, há um campo para que o profissional possa identificar se a pessoa possui ou não suporte familiar, bem como o estágio de motivação para a mudança naquele momento.

A seção exame físico foi composta por exame cardiorrespiratório, mensuração de altura e peso para classificação do Índice de Massa Corporal, circunferência abdominal, aferição da pressão arterial e glicemia capilar, além da avaliação dos pés.

Os pressupostos do autocuidado apoiado, um dos pilares do MACC, têm como base a metodologia dos “5 As” (avaliação, aconselhamento, acordo, assistência e acompanhamento)^(5)^. Desse modo, a seção 1 do instrumento oferece suporte para o enfermeiro avaliar as crenças, valores, conhecimentos, comportamentos dos indivíduos e identificar as necessidades de saúde objetiva e subjetiva, com esta na perspectiva do profissional e da pessoa.

O levantamento de problemas dentro da CE foi realizado buscando identificar as principais dificuldades relatadas pelos participantes em relação ao autocuidado e em hábitos adotados por meio de abordagem acolhedora, permitindo que o paciente se sentisse confortável para expressar suas dificuldades.

Ainda na seção 1 do instrumento, há espaço para o registro do acordo realizado entre o profissional e o usuário por meio da pactuação de metas, a partir do “problema” selecionado pelo usuário. Essa pactuação envolve os comportamentos e atividades que serão assumidos nos próximos dias e contribuir para responsabilização da pessoa com o processo e seus resultados. Para isso, é necessário que a pessoa consiga descrever o que fará, por quanto tempo, quantas vezes no dia ou na semana, aonde/quando e como, de forma específica e realista. Além disso, há a representação de um contrato, no qual o paciente deve assinar, com o objetivo de fomentar a responsabilização da pessoa com o processo e seus resultados (Figura 2).

**Figura 2:** Ilustração do plano de cuidados, plano de manutenção e contrato

A seção 2 do instrumento foi composta por questões de monitoramento das metas pactuadas que foram utilizadas nas ligações telefônicas entre as consultas presenciais. Esta teve como base a “assistência” e o “acompanhamento”, o qual tem como objetivo monitorar soluções implementadas por meio do plano de manutenção. O monitoramento telefônico se iniciava relembrando a meta que havia sido pactuada na consulta presencial. O usuário descrevia quais as dificuldades e facilidades foram enfrentadas no período, e era realizada a identificação de barreiras, sendo discutidas as possibilidades para superá-las.

A seção 3 foi composta pela estrutura de registo da consulta 1 para ser utilizada para novas pactuações, com esta tendo objetivo de “acompanhamento” e “assistência”. Quando as metas estabelecidas anteriormente eram alcançadas, era solicitado ao indivíduo que indicasse outro problema ou dificuldade a ser superada e as possíveis ações para contorná-la.

A seção 4 teve como objetivo a avaliação clínica final da intervenção, bem como de realizar o estímulo à mudança de comportamento nos participantes de forma duradoura.

A organização do instrumento em quatro seções teve como objetivo sistematizar o processo de cuidado para garantir um acompanhamento contínuo e estruturado da evolução dos participantes ao longo da intervenção.

## RESULTADOS

### Organização dos instrumentos

Os instrumentos utilizados foram construídos por enfermeiras pesquisadoras, a partir de orientações contidas na cartilha “Autocuidado Apoiado: manual do profissional de saúde”, constituída por quatro impressos para condução e registro das consultas de enfermagem (Quadro 1).

**Quadro 1:** Estruturação do instrumento

Instrumento	Objetivo
**Consulta de enfermagem inicial**	Levantar identificação, dados clínicos, comportamentais e sociais para diagnóstico situacional e planejamento do cuidado.
**Consulta de acompanhamento**	Monitorar a adesão às metas pactuadas, revisar o plano de autocuidado e renegociar estratégias.
**Monitoramento telefônico**	Avaliar a evolução das mudanças de comportamento e oferecer suporte para dificuldades encontradas.
**Consulta final**	Comparar os dados iniciais e finais, avaliando os impactos da intervenção na saúde do participante.

A partir dos pressupostos do autocuidado apoiado, o mesmo visa reorganizar as práticas assistenciais em contraposição à atenção biomédica predominante que não têm mostrado impactos efetivos no campo das condições crônicas. Baseia-se na premissa do empoderamento e preparação dos indivíduos para o autogerenciamento da sua condição de saúde^(5)^.

Ao fim da elaboração do instrumento, foi promovida uma discussão entre integrantes do grupo de pesquisa e profissionais que, no período da investigação, atuavam no serviço de saúde, com o objetivo de verificar a possibilidade de sua aplicação. Após discussão, foram necessários ajustes no instrumento por conta de sua extensão.

### Utilização do instrumento para sistematização da consulta de enfermagem

A condução das consultas, com duração média de cinquenta minutos, envolveu a aplicação de questionários auxiliares da pesquisa e coleta de dados antropométricos. Durante esse processo, a pesquisadora principal percebeu o estranhamento dos participantes ao serem questionados detalhadamente sobre hábitos de vida, ao serem ouvidos e estimulados a refletir sobre seus próprios hábitos.

A sistematização da CE, utilizando as premissas do autocuidado apoiado, permitiu o registro e aconselhamento de acordo com a individualidade e motivação de cada participante, o que favoreceu o aceite e reconhecimento da necessidade de mudança de comportamento, podendo ser verificado nas falas dos participantes durante o telemonitoramento e nos encontros presenciais na unidade.

A aplicação do instrumento estruturado facilitou as avaliações de comportamentos atuais, favorecendo a identificação, pelos usuários, dos hábitos necessários de mudanças. A partir da autoavaliação do comportamento atual, foi possível que a pesquisadora reconhecesse detalhes sobre a rotina dos participantes e esclarecesse possíveis dúvidas, o que potencializa o engajamento no processo de cuidado. A Figura 3 ilustra o plano de cuidados elaborado a partir da identificação de problemas identificados em conjunto pelo usuário e profissional.

**Figura 3:** Ilustração do plano de cuidados de acordo com os problemas identificados pelo usuário e profissional

É importante ressaltar que, a partir desses reconhecimentos, o enfermeiro organiza o “aconselhamento” utilizando as estratégias mais efetivas, apoiando o autocuidado e o processo de mudança de comportamento centrado na pessoa, na sua disponibilidade e na sua necessidade.

Inicialmente, os participantes demonstraram dificuldade em refletir sobre a necessidade mudança de comportamento. Além disso, havia a crença de que realizar o tratamento medicamentoso seria suficiente para o manejo da condição crônica, devido à sua familiaridade com o modelo biomédico. Isso resultou em uma resistência inicial ao tratamento não medicamentoso. No entanto, após a observação dos resultados clínicos decorrentes das mudanças de comportamento, os participantes passaram a reconhecer o impacto positivo dessas alterações.

Ressalta-se a necessidade de identificar o grau de confiança que a pessoa descreve ao propor determinada meta, visto que o grau de confiança mostra o quanto a pessoa acredita ser capaz de alcançar seus objetivos e sustentá-los. Para isso, ela precisa avaliar o esforço necessário para agir diferente. A Figura 4 apresenta a ilustração utilizada para o registro dessa informação.

**Figura 4:** Ilustração do grau de interesse na mudança de comportamento

Desta forma, logo após a pactuação, foi necessário dar uma nota de zero a dez para a confiança em conseguir executar o plano. O último item presente no primeiro impresso do instrumento é o contrato.

Ao fazer as pactuações, o profissional explica o objetivo do contrato e a importância da sua assinatura neste momento. Seu caráter é moral, reforçando o compromisso assumido consigo mesmo para a mudança^(6)^. Para isso, houve o questionamento referente aos aspectos positivos e negativos do processo de mudança. As soluções adequadas ao propósito devem ser reforçadas, e as inadequadas ou de maior dificuldade devem ser revistas e reorganizadas.

Além disso, à medida que uma tarefa foi concluída, outras são eleitas para que a habilidade em resolver problemas se desenvolva progressivamente com maior espontaneidade e autonomia. Em todos os impressos de consulta, existem os campos de realização de exame físico para que os parâmetros possam ser comparados.

A sistematização, por meio de instrumento específico (Figura 5), facilitou a condução da CE e favoreceu o registro das informações, garantindo, assim, uma documentação precisa, essencial para a comunicação interprofissional e para a continuidade do cuidado. Através da sistematização da consulta, foi possível realizar o acompanhamento contínuo da evolução do participante ao longo do tempo, permitindo ajustes no plano de cuidados conforme necessário.

**Figura 5:** *QR Code* para visualização do instrumento desenvolvido

Ao final da intervenção, observaram-se diferenças na autopercepção sobre a necessidade de mudança de comportamentos, além da melhoria de parâmetros clínicos. Essas modificações foram verificadas na fala dos participantes, nos dados laboratoriais e nos dados antropométricos coletados ao longo do período da pesquisa.

Diante desse cenário, o enfermeiro tem que lançar mão de estratégias para isso. As metas iniciais precisam ser tangíveis, pois, caso contrário, pode se sentir desmotivado e desistir, o que torna essa tarefa um desafio.

Dessa forma, a escuta ativa é essencial para captar queixas explícitas e identificar problemas que possam não ser mencionados diretamente, e a identificação das dificuldades do paciente é essencial para oferecer orientações eficazes e personalizadas. Ademais, durante o exame físico e a interação com o paciente, o enfermeiro pode identificar sinais que indiquem dificuldades no autocuidado, como higiene inadequada, alterações na pele e expressões faciais que demonstram desconforto ou insegurança.

## DISCUSSÃO

Para garantir a qualidade da assistência de enfermagem às pessoas com diabetes, o enfermeiro deve acompanhá-las mediante a realização da CE, baseada no processo de enfermagem, que objetiva conhecer a história pregressa da pessoa durante a coleta de dados subjetivos, a identificação de problemas existentes, o desenvolvimento de um plano assistencial direcionado, a priorização de diagnósticos de enfermagem, os resultados esperados e sua respectiva evolução^(6)^.

As práticas de saúde e de enfermagem ainda persistem com características do modelo biomédico, mecanicista, com foco no profissional, em detrimento de um agir em saúde que seja capaz de apreender as necessidades mais abrangentes dos usuários e famílias, em um contexto que vise um cuidado individualizado^(2,5)^.

A caracterização dos participantes tem como objetivo identificar as especificidades e necessidades individuais da pessoa, para que as ações estejam de acordo com necessidades sociais, além de intervir na realidade de forma articulada e corresponsabilizada^(7)^. Estudos demonstram que a CE e as ações educativas baseadas no autocuidado apoiado impactam a autogestão da saúde, aumentam o estímulo ao autocuidado e contribuem com a mudança de comportamento^(2,4,5)^.

Dessa maneira, é necessária a implementação de ações individuais e coletivas que possam estimular a conscientização dos indivíduos em reconhecer seu papel para o adequado manejo da doença no contexto dos serviços de saúde que compõem a APS e manutenção da qualidade de vida^(2,5)^. Vale salientar que essas ações demandam tempo, principalmente pela necessidade de o profissional compreender e aprender a trabalhar com as subjetividades dos usuários, que tem como base uma relação de confiança, e do paciente entender que o profissional constitui o apoio no processo de mudança de comportamento e engajamento com sua condição de saúde. Para tanto, os enfermeiros podem utilizar técnicas como entrevistas motivacionais, estabelecimento de metas realistas, *feedback* positivo e suporte contínuo ao paciente. O uso de diários de autocuidado e acompanhamento periódico também são estratégias eficazes para manter a motivação a longo prazo.

Em 1982, pesquisadores descreveram cinco diferentes estágios motivacionais para a mudança de comportamento: pré-contemplação; contemplação; preparação; ação; e manutenção. Por ser processo, é dinâmico, modificando-se no tempo e nas diferentes circunstâncias. A motivação, portanto, é um estágio interno de disposição para a mudança e sofre influência de fatores externos. A identificação do estágio de mudança é importante pois possibilita saber o quanto a pessoa está motivada para efetivamente mudar^(8)^. A motivação para a mudança engloba os estágios que precisam ser identificados para que as intervenções mais efetivas sejam implementadas. A avaliação desses estágios é decisiva para a escolha de estratégias terapêuticas mais adequadas e eficazes para o aumento e manutenção da motivação, favorecendo a mudança efetiva de comportamento.

Desse modo, a CE, quando realizada de forma sistematizada, constitui o cenário ideal para o estímulo ao autocuidado, contribuindo para a mudança de comportamento. Embora seja preconizada como atividade integrante da APS, a CE às pessoas com condições crônicas ainda não é sistematizada na maioria dos serviços de saúde no Brasil e, mesmo quando realizada, apresenta lacunas em sua implementação e execução^(9)^.

A assistência de enfermagem às pessoas com condições crônicas necessita ser voltada a um processo de educação em saúde que auxilie o indivíduo a conviver e reconhecer seu papel em relação ao manejo da condição crônica, tornando-o corresponsável por seu cuidado^(2,4)^. Estudo de meta-análise, realizado a partir de seis estudos clínicos randomizados, que objetivou determinar a eficácia das estratégias de autogestão para indivíduos com DM2, apontou redução da HbA1C nos grupos de intervenção, indicando a melhoria do tratamento ao contribuir com qualidade de vida e comportamento de autocuidado^(10)^.

No contexto brasileiro, a utilização do autocuidado apoiado durante a CE foi testada por estudo realizado na região Sul do Brasil, a qual demonstrou que, após avaliação da intervenção individual baseada no autocuidado apoiado (composta por CE e monitoramento telefônico), houve aumento significativo do conhecimento e da atitude positiva diante da doença, do impacto na qualidade de vida e da adesão às atividades de autocuidado^(2)^.

Contudo, alguns desafios permeiam a implementação da CE com esse enfoque, como as dificuldades dos pacientes com relação à reflexão sobre seu estilo de vida, porque esse processo leva tempo, requerendo um certo grau de autoconhecimento e de entendimento em relação ao seu próprio comportamento no dia a dia. O indivíduo necessita perceber que não é apenas a conduta medicamentosa que vai solucionar o problema, e esta resistência é morosa, além de ser influenciada pelos determinantes sociais, fatores socioeconômicos, educacionais e culturais que influenciam a percepção e adesão às mudanças comportamentais. Por exemplo, indivíduos com baixo nível educacional podem ter dificuldades em compreender orientações sobre autocuidado, enquanto fatores econômicos podem limitar o acesso a uma alimentação adequada e recursos para monitoramento glicêmico.

Para superar esses desafios, seria fundamental a implementação de diretrizes padronizadas para a CE, a capacitação contínua dos profissionais de enfermagem e o fortalecimento de políticas públicas que incentivem a consulta sistematizada e acessível a toda a população.

### Limitações do estudo

Como limitação, destaca-se a não utilização de sistema de linguagem padronizada durante a consulta. Reconhece-se que a utilização desta é fundamental para o processo de enfermagem e a sistematização da consulta, evidenciando a necessidade de investigações futuras que possam abordá-la de forma mais abrangente. Contudo, os resultados gerados a partir deste estudo podem contribuir para melhoria na qualidade do atendimento prestado pelo enfermeiro, além de identificar diversos aspectos da saúde da pessoa, incluindo não apenas aspectos físicos, mas também aspectos emocionais, sociais, familiares e psicológicos, promovendo uma abordagem integral do cuidado.

### Impactos para a prática assistencial de enfermagem

Destaca-se a importância da utilização de um instrumento auxiliar para o planejamento, sistematização e registro das ações de cuidado durante a CE às pessoas com diabetes. Essa prática não apenas facilita a organização e efetividade das intervenções, mas também promove uma assistência segura e de maior qualidade, contribuindo para a promoção da saúde e o manejo da condição crônica. Dessa forma, a implementação de estratégias de sistematização, como a realizada no presente estudo, pode contribuir para otimizar o processo de cuidado e alcançar resultados clínicos e de bem-estar mais positivos para essa população específica.

## CONSIDERAÇÕES FINAIS

A experiência da CE baseada no autocuidado apoiado emerge como uma ferramenta capaz de promover uma intervenção centrada no usuário, viabilizando a autonomia dos indivíduos enquanto mantém a essência cuidadora inerente à prática da enfermagem. A sistematização e o registro desse processo não apenas facilitam a adesão às mudanças necessárias em seu estilo de vida, mas também promovem o aprendizado e aumentam sua autoeficácia para lidar com os desafios do cotidiano. Assim, evidencia-se a relevância desse enfoque na prestação de cuidados de enfermagem, pois não apenas fortalece a relação terapêutica, mas também potencializa os resultados positivos na promoção da saúde e no bem-estar dos indivíduos.

## Data Availability

Os dados da pesquisa estão disponíveis no corpo do artigo.
